# Enzymatic Synthesis of Lysophosphatidylcholine Containing γ-Linolenic and Stearidonic Acids in a Solvent-Free System

**DOI:** 10.3390/foods15162914

**Published:** 2026-08-20

**Authors:** Matías Rivera-Báez, Fabrizzio Valdés-Rebolledo, Miguel Ángel Rincón-Cervera

**Affiliations:** 1 Institute of Nutrition and Food Technology, University of Chile, Santiago 7830490, Chile; matias.rivera@inta.uchile.cl (M.R.-B.); fabrizzio.valdes@veterinaria.uchile.cl (F.V.-R.); 2 Food Technology Division, ceiA3, University of Almería, 04120 Almería, Spain

**Keywords:** structured lipids, lysophosphatidylcholine, *Echium plantagineum*, γ-linolenic acid, stearidonic acid, biocatalysis, response surface methodology

## Abstract

Stearidonic acid (SDA) and γ-linolenic acid (GLA) are recognized for their anti-inflammatory and cardiometabolic benefits, yet their natural dietary sources remain scarce. This study aimed to develop and optimize the enzymatic synthesis of lysophosphatidylcholine (LPC) enriched with GLA and SDA in a solvent-free system using *Echium plantagineum* seed oil as the source of GLA and SDA. Enzyme screening was conducted with three immobilized lipases, among which Lipozyme^®^ 435 exhibited superior performance, achieving significantly higher proportions of both GLA and SDA into the LPC backbone compared with alternative lipases. Reaction optimization was performed using a Box–Behnken response surface design, evaluating temperature, time, lipase load, and substrate molar ratio. The analysis identified lipase load as the most influential factor for both fatty acid proportion and LPC yield. At 50 °C for 24 h, with a substrate molar ratio of 1:15 and a 15 wt% lipase load, the synthesis yielded 92.0 ± 4.1 mol%. Within the LPC fraction, GLA + SDA contributed 69.5 ± 1.2% of total fatty acids. The resulting structured LPC combines the enhanced bioavailability of the lysophospholipid carrier with the complementary metabolic activities of GLA and SDA, supporting its potential for the development of functional foods and nutraceutical formulations.

## 1. Introduction

Polyunsaturated fatty acids (PUFAs) are key nutrients for human health, with linoleic acid (LA, C18:2 n-6) and α-linolenic acid (ALA, C18:3 n-3) being essential PUFAs because they cannot be synthesized endogenously by the human organism and must be provided through the diet [1]. Beyond their intrinsic value, LA and ALA are direct precursors of γ-linolenic acid (GLA, C18:3 n-6) and stearidonic acid (SDA, C18:4 n-3), respectively, which have attracted growing scientific interest as they bypass the rate-limiting Δ6-desaturation step in PUFA metabolism, serving as efficient precursors for potent anti-inflammatory lipid mediators that attenuate chronic inflammatory conditions [2]. Dietary GLA is rapidly converted endogenously into dihomo-γ-linolenic acid (DGLA, C20:3 n-6), which yields anti-inflammatory eicosanoids [3]. Similarly, SDA acts as a highly efficient precursor of longer-chain n-3 PUFAs such as eicosapentaenoic acid (EPA, C20:5 n-3), thereby contributing to the production of pro-resolving mediators and modulating transcriptional regulators involved in lipid homeostasis [4].

While ALA is abundant in several vegetable seed oils such as those from chia (*Salvia hispanica* L.), flaxseed (*Linum usitatissimum* L.), or basil (*Ocimum basilicum* L.), natural sources of GLA and SDA are scarce. The seed oil of *Echium plantagineum*, a plant belonging to the Boraginaceae family, is currently one of the few commercially available sources of both PUFAs. It provides a balanced profile containing ~11% GLA and ~14% SDA of total fatty acids, alongside ~35% ALA [5,6]. Consequently, *E. plantagineum* seed oil has gained attention not only as an ingredient in cosmetics and personal care products but also as a dietary strategy to reduce cardiovascular risk, regulate inflammation and immune responses, mitigate age-related degenerative conditions, and regulate emotional disorders [7].

To maximize the physiological benefits of PUFAs, research is shifting from bulk oils to structured lipids (SLs). SLs are customized molecules, typically built on glycerol or phosphoglycerol backbones, in which native FAs are replaced with alternative ones to achieve specific nutritional or technological functionalities. While SLs can be produced via chemical synthesis, enzymatic methods are commonly preferred for food and pharmaceutical applications due to their high regioselectivity, mild reaction conditions, and lower environmental impact [8,9]. These enzymatic approaches help preserve FA integrity and reduce the formation of undesirable by-products, enabling the development of tailored lipids for a wide range of applications. Particularly, SLs as functional food ingredients with health-promoting functions are attracting increasing attention. The performance of SL synthesis reactions free of organic solvents aligns with green chemistry principles, preventing toxic residue contamination in the final product, which is a critical factor for food-grade ingredients [10].

Research on SLs has focused on triacylglycerols (TAG), particularly those containing medium-chain FAs (MCFAs) at the *sn*-1,3 positions of the glycerol backbone and a long-chain fatty acid (LCFA) at the *sn*-2 position. These are called MLM-TAG molecules and display distinctive digestive behavior, as they are rapidly hydrolyzed by pancreatic lipase in the intestine, enhancing lipid utilization compared with conventional dietary TAG composed primarily of LCFA [11].

In contrast, although significant breakthroughs have recently been achieved regarding the synthesis of structured phospholipids (PLs) and lysophospholipids (LPLs) [12,13], this area remains a promising yet underexplored research topic. PLs are amphiphilic molecules essential for membrane integrity and a wide range of cellular functions, including signaling, apoptosis, and protein regulation [14,15,16]. Although they constitute only a small proportion of dietary lipid intake, PLs exhibit high bioavailability and are efficiently incorporated into cell membranes, where they alter fatty acid composition, influence membrane biophysics, and modulate the formation of lipid-derived messengers such as eicosanoids, ultimately affecting inflammatory responses [14,17,18]. Beyond their physiological roles, PLs also hold significant industrial relevance as effective emulsifiers, antioxidants, and carriers for bioactive compounds [19].

Among PL derivatives, lysophosphatidylcholine (LPC) is particularly relevant. Unlike intact PL, dietary LPC (particularly *sn*-1-acyl-2-lysoPC) can be absorbed directly by the intestinal epithelium without the requirement for hydrolysis by phospholipase A2 (PLA2) [20], suggesting that it may serve as a more efficient delivery vehicle for bioactive FAs compared to both TAGs and PLs [21]. Furthermore, LPC is widely used as an emulsifier in food and feed formulations and improves lipid absorption [22], enhances carotenoid absorption [23], exhibits anti-inflammatory and antioxidant activities [24], and shows inverse associations with obesity [25,26,27].

Despite their potential advantages, there are currently no reports on the synthesis of structured LPC enriched with GLA and SDA. Therefore, this study aims to develop and optimize the enzymatic synthesis of structured *sn*-1-acyl-2-LPC containing GLA and SDA in a solvent-free system, using a response surface methodology approach. By combining the beneficial health properties of GLA and SDA with the superior bioavailability of the LPC carrier, this strategy seeks to generate novel functional food ingredients. Additionally, the resulting compounds may serve as food-grade emulsifiers, enhancing organoleptic properties and improving the intestinal absorption of lipophilic compounds.

## 2. Materials and Methods

### 2.1. Materials

*Echium plantagineum* seed oil (15200 NEWmegaTM Echium oil) was purchased from DeWit Specialty Oils (Texel, The Netherlands), and *sn*-glycero-3-phosphocholine (GPC, ≥95% purity, CAS Number 28319-77-9) was sourced from Cayman Chemical (Ann Arbor, MI, USA). Immobilized lipases from *Rhizomucor miehei* (Lipozyme^®^ RM-IM, activity 275 IUN/g according to the manufacturer), *Thermomyces lanuginosus* (Lipozyme^®^ TL-IM, activity 360 IUN/g according to the manufacturer), and recombinant *Candida antarctica* lipase B (Lipozyme^®^ 435, activity 9000 PLU/g according to the manufacturer) were kindly donated by Novozymes A/S (Bagsvaerd, Denmark). Unless otherwise stated, all other solvents and reagents used in this work were from Merck (Darmstadt, Germany).

### 2.2. Enzymatic Synthesis of LPC

#### 2.2.1. Lipase Screening

The suitability of Lipozyme^®^ RM-IM, Lipozyme^®^ TL-IM, and Lipozyme^®^ 435 as biocatalysts for the enzymatic synthesis of structured LPC was evaluated using two response variables: (i) the GLA + SDA proportion in the LPC fatty acid profile, and (ii) the LPC/PC molar ratio in the reaction products.

*E. plantagineum* seed oil was first hydrolyzed to free fatty acids (FFA) following the procedure described in our previous work [28]. The resulting hydrolysate was used as a substrate, together with GPC, for LPC synthesis via enzymatic esterification. Preliminary one-factor assays were conducted with each lipase individually to evaluate their suitability as biocatalysts. Reactions were performed in triplicate at 50 °C for 48 h in a solvent-free system, using a lipase load of 10 wt% relative to total substrates and a GPC:FFA molar ratio of 1:10 (0.2 mmol GPC and 2.0 mmol FFA). Syntheses were carried out in 10 mL conical-bottom Reacti-Vial™ glass vials using a Reacti-Therm™ III heating and stirring module (Thermo Scientific, Waltham, MA, USA).

After each reaction, a 20 mg aliquot of the product mixture was collected, and lipid classes were separated by thin-layer chromatography (TLC) on preparative silica gel plates (Silica gel 60, 0.5 mm, 20 × 20 cm) (Macherey-Nagel GmbH, Dueren, Germany) previously activated at 105 °C for 1 h. The mobile phase consisted of chloroform:methanol:water 65:25:4 (*v*/*v*/*v*). After development, lipid bands were visualized by exposure to iodine vapor under nitrogen and identified based on their retention factors.

LPC and PC bands were scraped from the TLC plate, and their FA profiles were determined by gas chromatography with flame ionization detection (GC-FID) as previously described [28]. Briefly, *n*-hexane (1 mL) was added to the silica-containing LPC or PC in 10 mL test tubes, and derivatization to FA methyl esters (FAME) was carried out using 2 mL of methanol:acetyl chloride (20:1 *v*/*v*) and heating the sealed tubes at 100 °C for 45 min. After cooling, distilled water was added, and the mixture was centrifuged at 3500 rpm for 5 min. The upper hexane layer was collected for GC-FID analysis in an Agilent 6890N GC system coupled to a 7683B autosampler (Agilent Technologies, Santa Clara, CA, USA) equipped with a Supelco SP-2560 capillary column (100 m × 0.25 mm × 0.2 μm). Initial temperature was set at 140 °C for 5 min, then increased at 4 °C/min to 190 °C, then at 1 °C/min to 220 °C, and then at 4 °C/min to 240 °C, with temperature kept at 240 °C for 5 min. Injector and detector temperatures were set at 270 and 260 °C, respectively, with nitrogen as the carrier gas at a flow rate of 1.9 mL/min and a split ratio of 1:100 (1 µL injection). Methyl tricosanoate (C23:0, purity ≥ 99.0%, Sigma-Aldrich, St. Louis, MO, USA) was added as an internal standard for quantification purposes. FAMEs were identified according to their retention times compared with analytical standards (Supelco 37 Component FAME mix). The LPC/PC molar ratio was calculated from the LPC and PC FA profiles as explained in a previous work [29].

#### 2.2.2. Reaction Optimization by Response Surface Methodology

A Box–Behnken response surface design comprising 27 experimental runs was applied to optimize the synthesis of structured LPC. The independent variables included the GPC:FFA molar ratio (1:5–1:15, using 0.2 mmol GPC and the proportional mmol amount of FFA in each case), lipase load (5–15 wt% relative to total substrates), reaction time (24–72 h), and temperature (40–60 °C). All reactions were performed using the equipment described previously. The response variables were the GLA + SDA proportion in the LPC FA profile after the enzymatic reaction and TLC separation, as well as the conversion yield, defined as the percentage of the GPC substrate that was converted to LPC.

Conversion yield was determined by quantifying the molar amount of LPC using ^31^P-Nuclear Magnetic Resonance (^31^P-NMR), with triphenylphosphate (TPP) as the internal standard. An aliquot of the reaction mixture (50 mg) was combined with 1 mL of a TPP solution (3 mg/mL) prepared in deuterated chloroform (CDCl_3_) and transferred to a 5 mm NMR tube. Spectra were acquired on a Bruker Avance NEO 400 MHz spectrometer (Billerica, MA, USA) under the following conditions: probe temperature 27 °C, 64 scans, 64 K data points, relaxation time 2 s, pulse width 8 µs, and acquisition time 0.5 s. The data treatment was performed in TopSpin 5.0.0 software (Bruker, Billerica, MA, USA). Conversion yield was calculated as follows:

(1) $$Yield\;\left(\mathrm{mol}\%\right)=\frac{Resulting\;LPC\;\left(\mathrm{mmol}\right)}{\;Initial\;GPC\;\left(\mathrm{mmol}\right)}*100$$

### 2.3. Separation of Synthesized LPC-Enriched Phospholipid Fraction

Following completion of the synthesis reaction under the optimized conditions (temperature, reaction time, lipase load, and substrate molar ratio), remaining FFA were removed by solid-phase extraction (SPE), loading an aliquot of the reaction mixture (200 mg) onto a Sep-Pak silica 6 cc Vac cartridge (1 g sorbent; Waters, Milford, MA, USA). Neutral lipids, including remaining FFA, were eluted with 25 mL of acetone:methanol 9:1 (*v*/*v*), after which polar lipids (primarily LPC) were recovered using 25 mL of pure methanol. The efficiency of the FFA removal was assessed by TLC on preparative silica gel plates (Alugram Xtra Sil G 5 × 10 cm) (Macherey-Nagel GmbH, Dueren, Germany). The mobile phase consisted of *n*-hexane:diethyl ether:acetic acid (80:20:1, *v*/*v*/*v*). After development, lipid bands were visualized by exposure to iodine vapor under nitrogen and identified based on their retention factors (Figure S1).

### 2.4. Synthesis and Separation of GLA- and SDA-Containing LPC-Enriched Fraction

LPC synthesis was performed under the optimal conditions identified for reaction time (24 h), temperature (50 °C), lipase load (15 wt%), and substrate molar ratio (1:15), using GPC and a GLA + SDA-enriched FFA concentrate as substrates. This concentrate was prepared by hydrolyzing *E. plantagineum* seed oil to obtain FFA, followed by enrichment of GLA and SDA through urea complexation, as previously described [28]. The resulting LPC was further separated by SPE and quantified by ^31^P-NMR using TPP as an internal standard according to the procedures outlined above. The ratios of *sn*-1-acyl-2-lysoPC to *sn*-1-lyso-2-acylPC and between LPC, as well as LPC to diacylated PC, were determined based on their characteristic signals in the NMR spectra (Figure S2).

### 2.5. Analysis of Oxidative Stability of the LPC-Enriched Fraction

The oxidative stability of the LPC-enriched fraction, the GLA + SDA FFA concentrate, and the original *E. plantagineum* seed oil was comparatively evaluated under accelerated storage at 50 °C for up to 72 h. Aliquots were sampled every 24 h to determine thiobarbituric acid reactive substances (TBARS) following a modified AOCS Cd 19–90 method. Briefly, 50 mg of each sample was solubilized in 1.0 mL of 1-butanol and mixed with 1.0 mL of 0.2% (*w*/*v*) TBA in 1-butanol. The mixture was incubated in a boiling water bath at 95 °C for 2 h and subsequently cooled in an ice bath. Absorbance was measured at 532 nm against a reagent blank. Quantification was performed using a standard curve prepared from a series of aliquots (0.1–1.0 mL) of 0.55 mM 1,1,3,3-tetraethoxypropane (≥96% purity) in 1-butanol. The results were expressed as mmol MDA/kg.

### 2.6. Statistical Analysis

All analyses were performed at least in triplicate, and results were reported as mean ± standard deviation. One-way analysis of variance (ANOVA) followed by Tukey’s post-hoc test was used to identify significant differences among values (*p* < 0.05) using StatGraphics Centurion 19 (Statgraphics Technologies, The Plains, VA, USA). The response surface methodology (Box–Behnken design) was carried out using Design-Expert 12 (Stat-Ease, Minneapolis, MN, USA).

## 3. Results

### 3.1. Lipase Screening

The hydrolyzed *E. plantagineum* seed oil used as substrate for LPC synthesis showed a predominance of unsaturated FA, mainly ALA (33.9% of total FA), LA (16.7%), oleic acid (OA, 16.2%), SDA (12.1%), and GLA (11.5%) (Table 1). Among the assayed enzymes, Lipozyme^®^ TL-IM exhibited the poorest performance as a biocatalyst, yielding a very low proportion of GLA (1.7% of total FA) and SDA (1.4%) in the LPC fatty acid profile, together with the lowest LPC/PC molar ratio (7.7) (Table 1). A high LPC/PC molar ratio is desirable, as it reflects a greater yield of the target product (LPC) relative to the PC by-product. Lipozyme^®^ RM-IM also showed limited efficiency for incorporating GLA (1.7%) and SDA (1.3%) into the GPC backbone, although it led to the highest LPC/PC molar ratio among the three assayed lipases (25.1). In contrast, Lipozyme^®^ 435 was the only biocatalyst able to generate a product mixture with significantly higher proportions of both GLA and SDA (11.7% each of total FA), while showing an intermediate value for the LPC/PC molar ratio (17.0). Based on its results, Lipozyme^®^ 435 was selected for subsequent optimization steps.

### 3.2. Optimization of Enzymatic LPC Production

A quadratic Box-Behnken response surface design with 27 experimental runs was used to identify the optimal combination of the values of reaction temperature (40–60 °C), time (24–72 h), lipase load (5–15 wt% of total substrates), and substrate molar ratio (1:5–1:15 mol/mol) to maximize the GLA + SDA proportion in the resulting LPC as well as the LPC synthesis yield. The results obtained are reported in Table 2.

#### 3.2.1. Optimized Response for the GLA + SDA Proportion in the LPC-Enriched Fraction

The ANOVA of the quadratic model showed that the overall model was highly significant (F-value = 7.85, *p*-value = 0.0005), indicating that the parameters included in the design substantially explain the variability in the GLA and SDA proportion in the LPC (Table 3). Among the main assayed variables, lipase load was the most influential one, showing that enzyme concentration plays a critical role in driving the synthesis of these SLs (F-value = 50.55, *p*-value < 0.0001). Temperature also exerted a significant influence on the response (F-value = 8.26, *p*-value = 0.0140). In contrast, reaction time and substrate molar ratio were not statistically significant.

Regarding factor interactions, most of them were not significant, with the only exception of time x substrate molar ratio (F-value = 7.04, *p*-value = 0.0211), suggesting a synergistic effect between these variables. As for the quadratic effect, only the quadratic term for time was significant (F = 26.98, *p*-value = 0.0002), which indicates a significant curvature in the response surface associated with reaction time. None of the other quadratic terms had significant effects.

The lack-of-fit test was not significant (*p*-value = 0.1103), confirming that the model adequately fits the experimental data. Thus, the response variable (GLA + SDA proportion in the LPC) was primarily influenced by the lipase load, followed by temperature, with a nonlinear effect of time and a significant interaction between time and substrate molar ratio.

**Figure 1:**
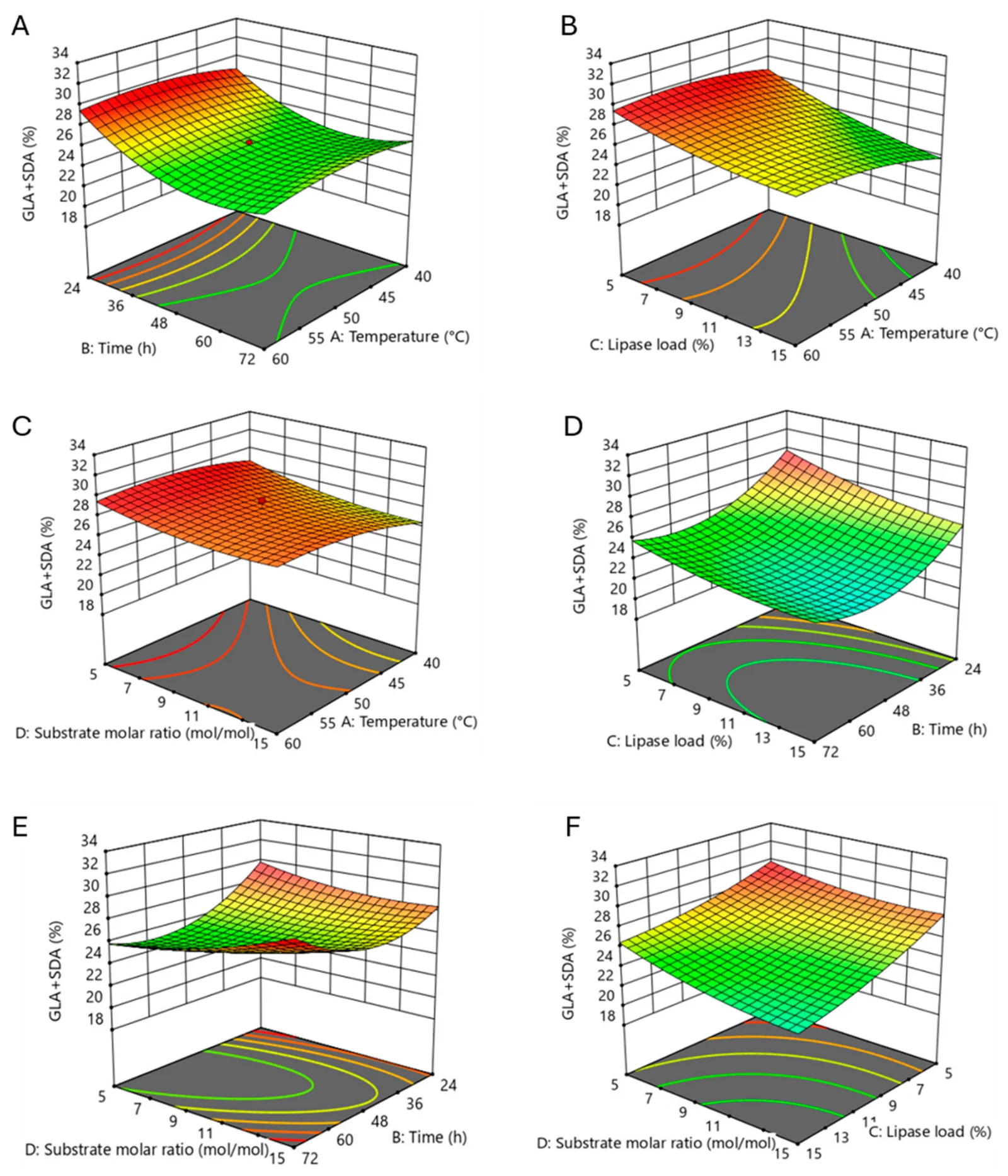
3D-response surface plots showing the influence of combined variables (reaction time, temperature, lipase load, and substrate molar ratio) on the GLA and SDA proportion in the enzymatic products (% of total FAs). (**A**) Time × Temperature plot; (**B**) Lipase load × Temperature plot; (**C**) Substrate molar ratio × Temperature plot; (**D**) Lipase load × Time plot; (**E**) Substrate molar ratio × Time plot; (**F**) Substrate molar ratio × Lipase load plot.

The three-dimensional response surface plots depicting the influence of the assayed factors (time, temperature, lipase load, and substrate molar ratio) on the GLA + SDA proportion in the resulting LPC are shown in Figure 1. Based on the model, the optimal reaction conditions were identified at 55 °C, 24 h, a lipase load of 5.0 wt% relative to substrates, and a substrate molar ratio of 1:5 to yield a predicted GLA + SDA of 29.8 ± 1.0% of total FAs.

#### 3.2.2. Optimized Response for LPC Synthesis Yield

The ANOVA indicates that the quadratic Box-Behnken model provides a statistically significant explanation of the variability in LPC synthesis yield (F-value = 8.00, *p*-value = 0.0005). Among the linear factors, lipase load (F-value = 38.94, *p*-value < 0.0001) and substrate molar ratio (F-value = 32.17, *p*-value = 0.0001) exerted a significant influence on the response (Table 4). Temperature showed a moderate but still significant effect (F-value = 6.05, *p*-value = 0.030), whereas reaction time did not contribute meaningfully under the tested conditions.

Interaction terms were not statistically significant, suggesting limited synergistic effects between pairs of variables. Regarding quadratic terms, both A^2^ (temperature) and C^2^ (lipase load) were significant, indicating curvature in the response surface for temperature and lipase load, while D^2^ (substrate molar ratio) is marginal and B^2^ (time) is negligible.

The non-significant lack-of-fit (*p*-value = 0.3814) confirms that the model adequately describes the experimental data, with lipase load and substrate molar ratio as the primary drivers related to LPC yield. However, the variability among center points reflects experimental dispersion that should be considered when interpreting robustness.

**Figure 2:**
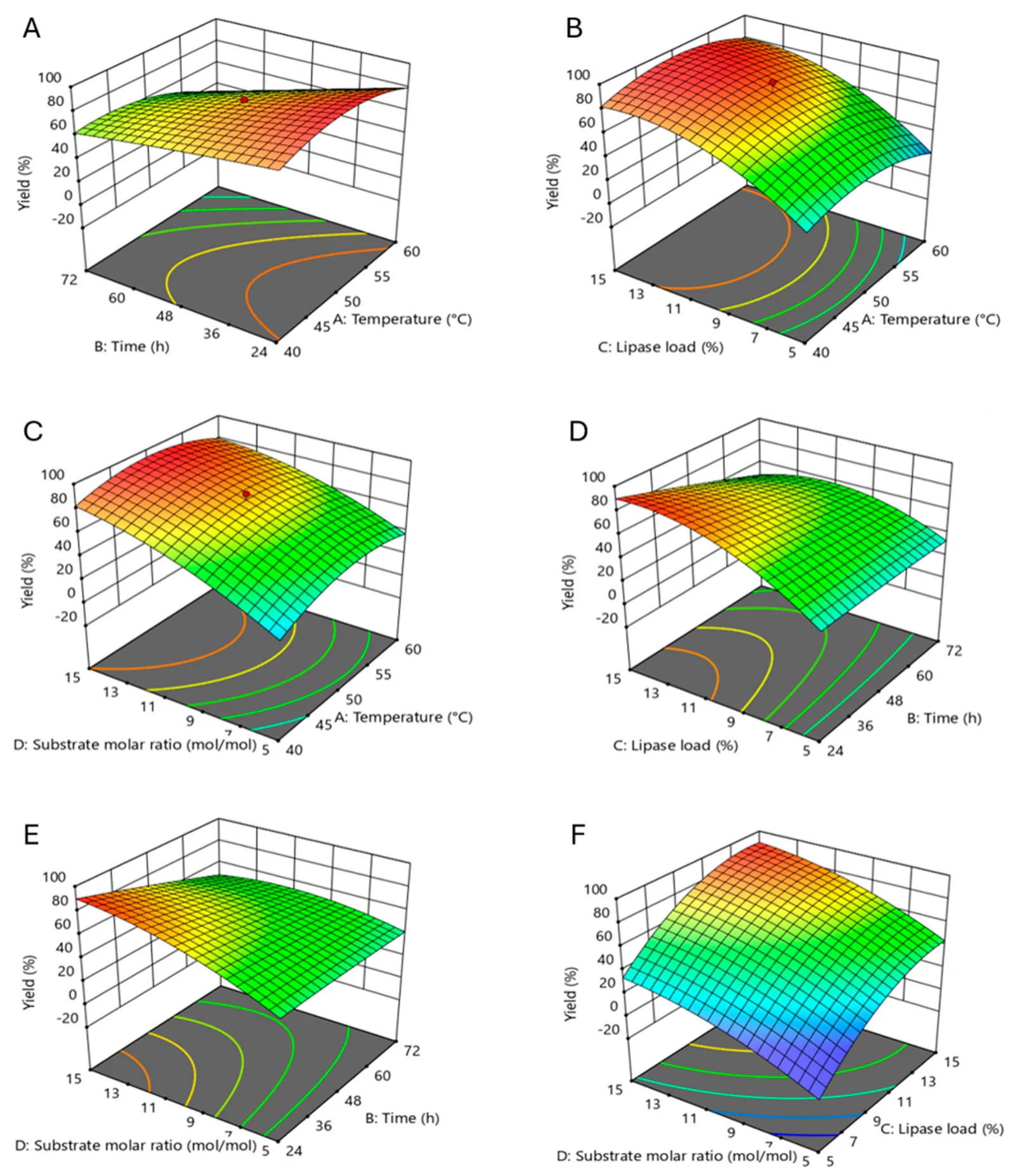
3D-response surface plots showing the influence of combined variables (reaction time, temperature, lipase load, and substrate molar ratio) on the LPC synthesis yield. (**A**) Time × Temperature plot; (**B**) Lipase load × Temperature plot; (**C**) Substrate molar ratio × Temperature plot; (**D**) Lipase load × Time plot; (**E**) Substrate molar ratio × Time plot; (**F**) Substrate molar ratio × Lipase load plot.

The 3D-response surface plots depicting the influence of the assayed factors on the response variable (LPC yield) are shown in Figure 2. According to the model, the highest predicted LPC yield conversion was 93.7 ± 4.5 mol% under the optimal conditions of 50 °C, 24 h, GPC: FFA 1:15 molar ratio, and a 15 wt% lipase load. Under these conditions, the predicted FA profile of the resulting LPC would present 22.5 ± 1.0% of GLA + SDA. To validate this prediction, an experiment was conducted applying the identified values of temperature, time, substrate molar ratio, and lipase load to maximize LPC yield, achieving a synthesis yield of 89.8 ± 2.3 mol% and an LPC FA profile containing 22.4 ± 0.3% GLA + SDA, which are within the expected ranges predicted by the response surface model (Table 5).

### 3.3. Synthesis and Separation of LPC-Enriched Fraction with GLA and SDA

In this study, yield maximization was prioritized, as the proportion of GLA and SDA in the LPC can be enhanced by using a PUFA concentrate from *E. plantagineum* seed oil instead of the hydrolyzed oil. The optimal conditions identified through the Box-Behnken experimental design (50 °C, 24 h, GPC: FFA 1:15 molar ratio, and a lipase load of 15 wt%) were applied. Under these conditions, an LPC yield of 92.0 ± 4.1 mol% and a proportion of GLA and SDA in LPC of 69.5 ± 1.2% were achieved (Table 5). Regarding the distribution of reaction products, the ratio of *sn*-1-acyl-2-lysoPC to *sn*-1-lyso-2-acylPC was 6.4, while the LPC/PC ratio reached 17.9, considering LPC as the sum of both *sn*-1-acyl-2-lysoPC and *sn*-1-lyso-2-acylPC.

### 3.4. Analysis of Oxidative Stability of the LPC-Enriched Fraction

**Figure 3:**
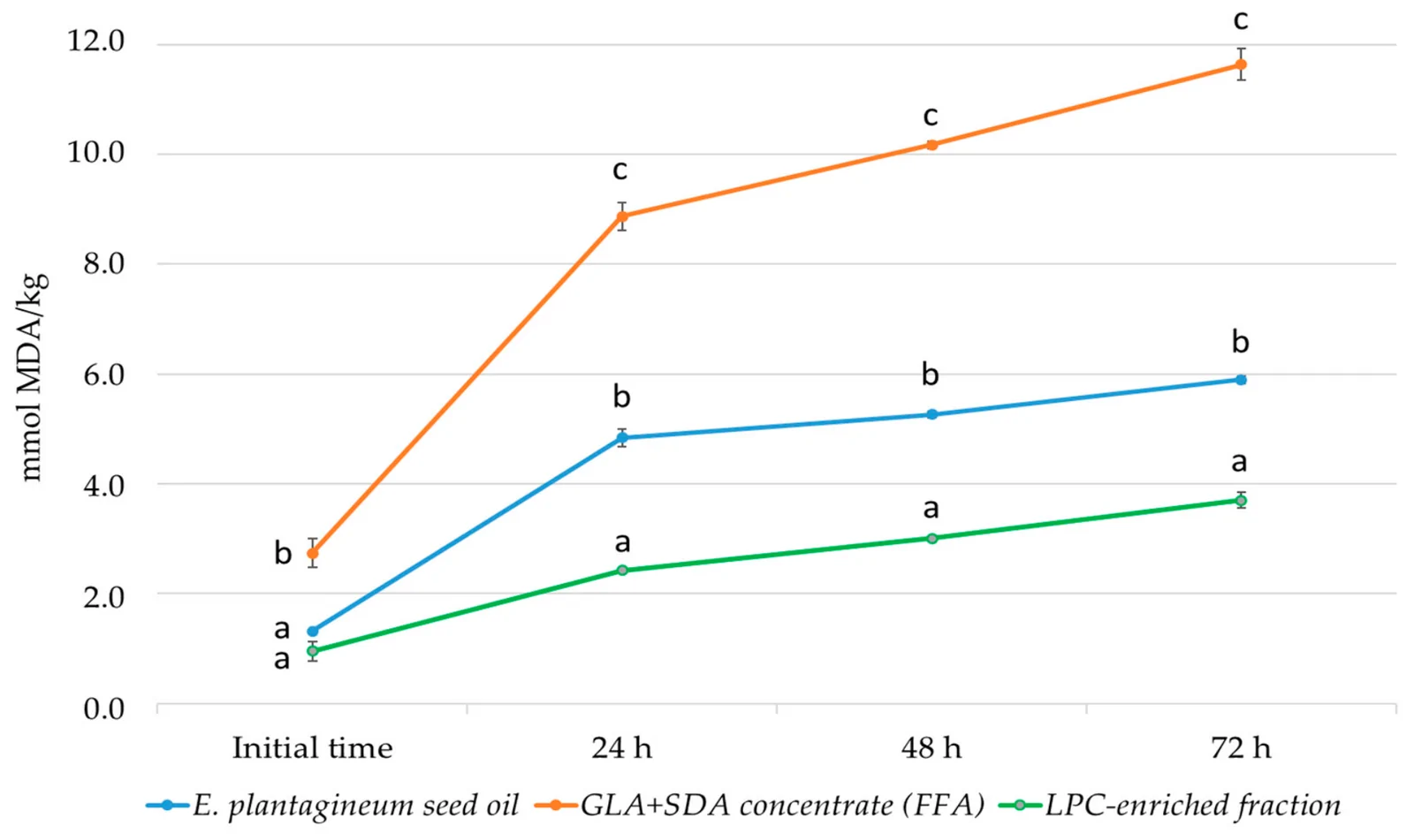
TBARS values (mmol MDA/kg) of *E. plantagineum* seed oil, the GLA + SDA concentrate as FFA, and the LPC-enriched fraction separated from the reaction products after being heated at 50 °C for 72 h. Values not sharing the same letter are significantly different (*p* < 0.05).

The evolution of TBARS values for the LPC-enriched fraction, the GLA and SDA concentrate as FFA, and the original *E. plantagineum* seed oil is shown in Figure 3. Significantly higher values were observed in the FFA concentrate at all time points, ranging from 2.74 to 11.64 mmol MDA/kg at the beginning of the experiment and after 72 h of heating at 50 °C, respectively. For *E. plantagineum* seed oil, values ranged between 1.32 and 5.90 mmol MDA/kg, whereas the lowest TBARS values were consistently found in the LPC-enriched fraction (0.95–3.70 mmol MDA/kg). These results indicate that PUFA undergo significantly less oxidation when esterified into the LPC backbone compared with their presence in common oils (TAG) or as FFA.

## 4. Discussion

### 4.1. Lipase Screening

The lipase screening for LPC synthesis containing GLA and SDA identified Lipozyme^®^ 435 as the most suitable biocatalyst to produce the target product (*sn*-1-acyl-2-lysoPC), outperforming Lipozyme^®^ RM-IM and Lipozyme^®^ TL-IM. Lipozyme^®^ 435 is an immobilized form of *Candida antarctica* lipase B (CALB) commercially available on a hydrophobic macroporous acrylic resin (Lewatit^®^ VP OC 1600, Lanxess, Pittsburgh, PA, USA). Although CALB is often regarded as non-regiospecific, several studies have reported *sn*-1,3 preference under particular conditions (e.g., during alcoholysis of acylglycerols with a large excess of ethanol, in direct esterification of glycerol to produce 1,3-diacylglycerols with specific solvents, and in interesterification of TAGs with fatty acid ethyl esters) [30]. CALB has also shown a tendency towards the *sn*-1 position in PL substrates under low-water or solvent-free conditions, which likely promotes formation of *sn*-1-acyl-2-lysoPC [31].

Because the three commercial immobilized lipases were compared under equal-mass loading, and their activities are reported in different units by the manufacturers, the present study does not allow conclusions about intrinsic catalytic efficiency or specificity. Instead, the results support the practical outcome that Lipozyme^®^ 435 consistently performed better than Lipozyme^®^ RM-IM and Lipozyme^®^ TL-IM under the tested solvent-free conditions. The comparatively lower yields obtained with Lipozyme^®^ RM-IM and Lipozyme^®^ TL-IM reflect their limited performance in this experimental setup, which may be influenced by tolerance to highly unsaturated PUFA substrates or sensitivity to reaction parameters such as water activity [10,32], but these mechanistic aspects were not directly evaluated here.

Lipozyme^®^ RM-IM is *Rhizopus miehei* lipase immobilized on Duolite A568, a weak anion-exchange resin, while Lipozyme^®^ TL-IM is *Thermomyces lanuginosus* lipase immobilized on granulated silica. The hydrophobic Lewatit VP OC 1600 support promotes interfacial activation of lipases like CALB by better exposing the active site and enhancing reaction performance, which may explain the higher activity of Lipozyme^®^ 435 in the production of GLA- and SDA-enriched LPC. In contrast, Duolite A568 provides weaker ionic interactions that may be less favorable for PUFAs, and the hydrophilic character of granulated silica limits the interfacial activation required for optimal reaction [33].

The LPC/PC molar ratio further indicated that secondary esterification reactions occurred to some extent, leading to the formation of the diacyl-PC. This outcome is consistent with previous reports describing progressive conversion of LPC to PC under extended reaction times or high enzyme loads [32,34]. These findings highlight that both enzyme specificity and substrate structure are critical determinants of reaction efficiency and product distribution.

Because only Lipozyme^®^ 435 among the three commercial lipases tested efficiently produced GLA- and SDA-enriched LPC, and considering that it is food-grade, this lipase could be considered a suitable choice for producing structured LPC enriched with GLA and SDA for nutritional or nutraceutical applications.

### 4.2. Optimization of Enzymatic LPC Production

The response surface analysis demonstrated that lipase load was the most influential factor affecting both GLA and SDA LPC production and LPC synthesis yield, which is consistent with the behavior of solvent-free systems where high viscosity limits mass transfer and enzyme availability becomes a key parameter [10,34]. Increasing enzyme concentration enhances substrate–enzyme interactions, thereby improving reaction efficiency.

Temperature exerted a significant but secondary effect, with an optimum near 50 °C. Temperature is considered a relevant factor regarding the thermodynamics and kinetics of enzyme-catalyzed reactions; although Lipozyme^®^ 435 has been reported to tolerate high temperatures (70–80 °C), practical operation is recommended between 40 and 60 °C to maximize efficiency while avoiding thermal inactivation and limiting oxidation or degradation of highly unsaturated FAs [35]. A reaction time of 24 h produced higher LPC yields than 48 and 72 h, suggesting that while esterification proceeds efficiently at early stages, extended reaction times favor competing reactions such as hydrolysis, acyl migration, and secondary esterification [34]. The substrate molar ratio significantly influenced LPC yield but had little effect on the FA profile. This pattern indicates that while excess FFA enhances overall conversion, the proportion of GLA and SDA is primarily determined by enzyme specificity and substrate composition. The relatively stable proportion of these PUFAs across experimental conditions supports this interpretation.

Non-linear and interactive dynamics proved decisive. In the esterification model, the quadratic term of time (B^2^) was highly significant, revealing that prolonged incubation enhances the incorporation of GLA and SDA only up to an optimal threshold. Similarly, in the yield model, quadratic effects of temperature (A^2^) and lipase load (C^2^) were significant, highlighting that excessive enzyme loading or thermal shifts can reduce efficiency. Interactions between variables were generally weak, though the significant time × substrate molar ratio effect (BD) in esterification and borderline interactions in the yield model point to potential synergistic relationships.

The non-significant lack-of-fit in both models validates the suitability of the quadratic Box–Behnken design for capturing experimental variability. Taken together, these findings demonstrate that lipase load is the dominant determinant of both esterification and yield, with temperature acting as a secondary factor, while time and substrate ratio contribute primarily through non-linear and interactive effects. These insights reinforce the importance of response surface methodologies to unravel complex parameter interdependencies and to define optimal conditions for enzymatic synthesis of structured LPC enriched with GLA and SDA.

Although the optimal values for substrate molar ratio and lipase load differed between the esterification and yield models, the proportion of GLA and SDA in LPC varied within a much narrower range than the synthesis yield. Given that the primary objective was to maximize LPC yield, the conditions identified for yield optimization were selected to assay LPC synthesis using the GLA + SDA concentrate as substrate.

### 4.3. Validation of Optimal Conditions and Effect of Using PUFA Concentrate

The optimized reaction conditions (50 °C, 24 h, 15 wt% enzyme load, 1:15 substrate molar ratio) produced an LPC yield of 92.0 ± 4.1 mol%, confirming the validity of the predictive model. This yield is comparable to values previously reported for enzymatic LPL synthesis under optimized conditions. For example, Wang et al. (2020) reported a 93.1 mol% LPC yield enriched in n-3 PUFA (EPA and DHA), with 89.4% n-3 PUFA proportion, in a solvent-free enzymatic esterification of GPC catalyzed by the immobilized lipase MAS1 (from marine *Streptomyces* sp. strain W007) [36]. Their optimum conditions (55 °C, 24 h, 1:20 substrate molar ratio) are similar to those used here. Liu et al. (2017) described the synthesis of LPC enriched in n-3 PUFA using Lipozyme^®^ TL-IM (45 °C, 15 wt% lipase, 1:20 GPC:n-3 PUFA, 64 h), achieving 74.1 mol% LPC yield and 80.4% EPA + DHA of total FAs [32]. Zhang et al. (2022) optimized the enzymatic production of lysophosphatidylserine (LPS) enriched in DHA with Lipozyme^®^ TL-IM, reporting an 88.98 mol% synthesis yield at 45 °C, 20 wt% lipase load and a 1:15 substrate molar ratio (reaction time not reported) [8]. Taken together, these studies and our work converge on comparable operating windows (temperature 45–55 °C, ca. 15–20 wt% lipase load, and substrate molar ratios of 1:15–1:20).

Although Lipozyme^®^ TL-IM has delivered good yields and FA incorporation for marine n-3 PUFA substrates (EPA and DHA), it performed poorly in our attempts to incorporate GLA and SDA, highlighting the strong effect of substrate specificity of lipases toward FA structure and degree of unsaturation. Moreover, regardless of lipase type, excessive acyl-donor concentration can inhibit lipase activity by acidifying the micro-aqueous environment around the enzyme. An acidic microenvironment increases electrophilicity at carbonyl carbons and promotes acyl migration from the *sn*-1 to the *sn*-2 position on the GPC backbone, increasing formation of *sn*-1-lyso-2-PC and PC [8,32]. Likewise, raising the reaction temperature above ~50–55 °C can accelerate side reactions and acyl migration, further favoring undesired isomerization and PC formation [8,32].

Using a GLA/SDA-enriched concentrate as the acyl donor markedly increased incorporation of these PUFAs into the GPC backbone. The enrichment raised the combined GLA + SDA proportion from 21.9% to 69.5% while maintaining a high overall LPC yield. This effect is consistent with reduced competition from other FAs, particularly ALA, and aligns with results reported for the enzymatic synthesis of other SLs selectively enriched with GLA and SDA such as 1,3-diacylglycerols [37].

### 4.4. Oxidative Stability of the LPC-Enriched Fraction

The analysis of secondary oxidation products, expressed as mmol MDA/kg lipids, suggested that the specific molecular structure of a lipid class plays a more critical role in oxidative stability than the total proportion of PUFA. Although both the LPC-enriched fraction and the FFA concentrate exhibited highly similar FA profiles (~33.0 mol% GLA and ~36.5 mol% SDA; Table 5), the TBARS values of the FFA concentrate were significantly higher than those of the LPC-enriched fraction across all assayed time points (Figure 3). FFA are highly susceptible to lipid peroxidation as they decrease local pH and actively catalyze the decomposition of lipid hydroperoxides into pro-oxidant free radicals [38]. Furthermore, unesterified acyl chains possess greater molecular mobility, leaving them highly exposed to radical attack. In contrast, the *Echium plantagineum* seed oil, which is predominantly composed of TAGs, showed intermediate oxidative degradation. Esterification of FAs to a glycerol backbone enhances baseline physical stability; however, the absence of polar functional groups renders the bulk TAG matrix moderately vulnerable to oxidation. In contrast, the LPC-enriched fraction exhibited superior oxidative stability that could be explained by the presence of a hydrophilic headgroup capable of forming protective colloidal micellar networks. These supramolecular structures effectively isolate vulnerable double bonds from radical initiators while simultaneously acting as chemical chelators that bind pro-oxidative trace metal ions [39]. Remarkably, this enhanced stability was observed despite the LPC fraction possessing a highly unsaturated FA profile virtually identical to that of the FFA concentrate. The protective outcome can therefore be attributed to the combined physical and chemical antioxidant mechanisms inherent to polar PL.

### 4.5. Nutritional, Functional and Technological Significance of GLA + SDA-Enriched LPC

*Sn*-1-acyl-2-LPC is considered an effective PUFA carrier, facilitating intestinal absorption through phospholipase A_2_-independent pathways and specific transport mechanisms such as the major facilitator superfamily domain-containing protein 2A (MFSD2A). These mechanisms may offer distinct advantages for PUFA delivery to peripheral tissues compared to triacylglycerol (TAG) or FFA forms under certain conditions [20]. LPC has also been shown to enhance the intestinal uptake of other dietary lipids, suggesting a role in enhancing its nutritional relevance [40]. Although enzymatic synthesis of *sn*-1-DHA-2-LPC has attracted considerable interest because of its reported brain bioavailability and reported effects on brain development and neuronal growth [41], fewer studies have focused on the production of PUFA-enriched LPC from terrestrial plant sources.

The production of GLA- and SDA-rich LPC may offer potential metabolic advantages based on the known physiological properties of these fatty acids. Both PUFAs bypass the rate-limiting Δ6-desaturase step in PUFA metabolism, which may improve their conversion into biologically active metabolites. GLA is rapidly elongated to dihomo-γ-linolenic acid (DGLA, C20:3 n-6), a precursor of anti-inflammatory eicosanoids, whereas SDA is more readily converted into EPA than α-linolenic acid, potentially contributing to improved n-3 PUFA status [3,6]. The simultaneous incorporation of GLA and SDA into LPCs could therefore provide complementary physiological effects by promoting anti-inflammatory responses while limiting excessive arachidonic acid accumulation [2], although these effects were not directly evaluated in the present study. In addition, *sn*-1-acyl-2-LPC is thermodynamically more stable than its regioisomer, *sn*-1-lyso-2-acyl-PC, with an equilibrium strongly favoring the former [42].

LPC also represents a dietary source of choline, the precursor of the neurotransmitter acetylcholine. Choline deficiency has been associated with impaired hepatic lipid metabolism, and although endogenous synthesis occurs in humans, it is generally insufficient to meet physiological requirements, making dietary intake particularly important during pregnancy and lactation [18]. Consequently, LPC enriched with nutritionally valuable PUFAs could represent an interesting multifunctional lipid ingredient, although in this particular case these nutritional effects would require confirmation through appropriate biological studies.

In addition to their nutritional relevance, LPC molecules possess amphiphilic characteristics that make them effective emulsifiers in food systems [43,44]. Their ability to stabilize oil–water interfaces suggests potential applications as functional food ingredients and as delivery vehicles for nutraceuticals. Likewise, LPC has been widely used as a feed emulsifier to improve lipid digestion and nutrient utilization in animal nutrition [45,46]. Although these applications were not assessed in the present work, the high synthesis yields achieved under solvent-free conditions may facilitate the production of food-grade SLs suitable for future food and feed applications [9,22,47].

From a technological perspective, the present results compare favorably with previous reports on enzymatic LPC synthesis. The yields obtained under solvent-free conditions were within or above the range reported for related enzymatic systems [30,34]. Differences among studies are likely attributable to variations in enzyme specificity, substrate composition, and reaction conditions.

The yields reported in this study also exceeded those described for several acidolysis-based LPC synthesis systems [34], possibly reflecting the higher reactivity associated with direct esterification and the reduced occurrence of competing reaction pathways. Furthermore, the solvent-free approach produced yields comparable to or greater than those obtained in solvent-based systems [35], suggesting potential advantages from both environmental and industrial perspectives. Eliminating organic solvents can simplify downstream purification, reduce environmental impact, and better align the process with the principles of green chemistry, which are particularly relevant for food and nutraceutical applications.

An interesting feature of this work is the use of PUFA-enriched substrates derived from *Echium plantagineum* seed oil, a sustainable plant-based source of n-3 and n-6 PUFAs. As a result, the synthesized LPC species represent plant-derived structured lipids with a composition that could be of nutritional interest. Although the potential health benefits of these molecules remain to be demonstrated experimentally, the proposed strategy provides a promising platform for producing tailored LPC species with defined fatty acid compositions, supporting future studies aimed at developing functional food ingredients, nutraceuticals, and specialized lipid delivery systems.

### 4.6. Process Constraints and Perspectives

Water activity is a critical parameter in solvent-free enzymatic esterification, as it directly influences the esterification/hydrolysis equilibrium in SL synthesis reactions [48]. In this study, a high molar excess of FFA relative to GPC was employed to provide a strong thermodynamic driving force and shift the equilibrium toward esterification according to the law of mass action, thereby counteracting the reverse hydrolysis triggered by the water released *in situ*. This phenomenon is well supported by previous reports on the solvent-free synthesis of structured LPC and other SLs [49,50].

Active water-removal strategies (e.g., molecular sieves, vacuum dehydration) were not applied here due to practical constraints; the reactions were conducted at mmol scale in sealed mini-reactors, where such measures could have impaired mass transfer. Moreover, complete water removal can compromise lipase stability by depleting the hydration shell required for enzymatic activity [51,52]. Even without external water control, the optimized 1:15 GPC:FFA molar ratio achieved high conversion, comparable to yields reported in the literature at higher substrate ratios [36]. This range is even conservative compared with studies employing ratios up to 1:20 or even 1:60, as reviewed previously [47]. In addition, the surplus FFA separated from the target products after the reaction can be recovered and repurposed for other applications, such as the manufacture of fatty alcohols, soaps, cosmetics, or biofuels [53,54].

Nevertheless, reliance solely on substrate excess has inherent limitations. Elevated GPC:FFA ratios increase raw-material consumption, particularly critical when costly PUFA concentrates are used, and impose a downstream purification burden. High FFA concentrations may also reduce lipase performance by increasing local acidity and viscosity, promoting acyl migration, and accelerating enzyme deactivation [52]. These trade-offs highlight the importance of optimizing substrate ratios to balance conversion efficiency, purification requirements, and enzyme reuse.

External water-control measures could provide meaningful benefits for industrial or continuous production [52]. Actively removing water during esterification may allow the equilibrium to be driven toward synthesis at lower substrate ratios, reducing PUFA donor consumption and easing purification. However, such strategies must be weighed against potential drawbacks, including added process complexity, capital costs, and possible enzyme destabilization. Future work directly comparing substrate-excess-only and water-controlled schemes, ideally with direct measurement of water activity, would help clarify whether the efficiency gains justify the additional operational burden at larger scale.

## 5. Conclusions

This study successfully optimized the enzymatic synthesis of LPC enriched with GLA and SDA in a solvent-free system using *Echium plantagineum* seed oil. Among the assayed biocatalysts, Lipozyme^®^ 435 exhibited superior performance, achieving a significantly higher proportion of both GLA and SDA into the resulting LPC than alternative immobilized lipases, highlighting its superior performance among the tested commercial immobilized preparations under equal-mass conditions.

The application of Response Surface Methodology allowed for the identification of optimal reaction conditions, with enzyme load emerging as the most influential factor for both FA incorporation into the LPC backbone and overall LPC yield.

The resulting LPC-enriched fraction combines improved bioavailability associated with the LPL carrier system with the complementary metabolic activities of its constituent FAs, suggesting strong potential for applications in functional foods and nutraceutical formulations. Particularly, this SL integrates anti-inflammatory and cardiometabolic benefits associated with GLA- and SDA-derived lipid mediators, while also offering useful emulsifying properties relevant for food applications. Overall, this work establishes a robust and sustainable platform for the design of value-added PL-based functional ingredients enriched in health-promoting PUFAs.

Future research should focus on validating the health potential of these SLs through targeted *in vitro* assays (e.g., cellular uptake, inflammatory and lipid-metabolism endpoints) and controlled *in vivo* models to assess bioavailability, metabolic fates, safety, and physiological effects. Such studies will be essential to substantiate functional claims and to guide formulation and dosage strategies for eventual food and nutraceutical applications.

## Figures and Tables

**Table 1:** FA profile (percentage of total FAs) of the *E. plantagineum* seed oil hydrolysate used as substrate and the LPC-enriched fraction synthesized using Lipozyme^®^ RM-IM, Lipozyme^®^ TL-IM, or Lipozyme^®^ 435 in one-factor assays, as well as the LPC/PC molar ratio in each case. Reactions were carried out at 50 °C for 48 h, using a lipase load of 10 wt% relative to total substrates, and a GPC:FFA molar ratio of 1:10. Within each row, different superscript letters indicate significantly different values (*p* < 0.05).

Fatty Acids	*E. plantagineum* Seed Oil Hydrolysate	LPC-Enriched Fraction		
		Lipozyme^®^ RM-IM	Lipozyme^®^ TL-IM	Lipozyme^®^ 435
C16:0	6.4	9.5 ± 0.9 ^a^	10.2 ± 0.4 ^a^	9.1 ± 0.8 ^a^
C18:0	3.1	5.0 ± 0.1 ^b^	4.7 ± 0.2 ^ab^	4.5 ± 0.1 ^a^
C18:1 n-9	16.2	24.4 ± 0.6 ^b^	27.0 ± 0.8 ^c^	19.4 ± 0.4 ^a^
C18:2 n-6	16.7	20.9 ± 0.1 ^c^	20.2 ± 0.1 ^b^	15.3 ± 0.4 ^a^
C18:3 n-6 (GLA)	11.5	1.7 ± 0.2 ^a^	1.7 ± 0.1 ^a^	11.7 ± 0.1 ^b^
C18:3 n-3	33.9	37.1 ± 0.4 ^c^	34.7 ± 0.2 ^b^	28.5 ± 0.0 ^a^
C18:4 n-3 (SDA)	12.1	1.3 ± 0.2 ^a^	1.4 ± 0.1 ^a^	11.7 ± 0.1 ^b^
LPC/PC molar ratio		25.1 ± 0.8 ^c^	7.7 ± 0.8 ^a^	17.0 ± 1.4 ^b^

**Table 2:** Box-Behnken design and experimental results of the GLA + SDA proportion in the resulting LPC and reaction yield.

Run	Temperature (°C)	Time (h)	Lipase Load (wt% of Substrates)	GPC:FFA Molar Ratio	GLA + SDA in LPC (% of Total FA)	LPC Yield (mol%)
1	50	72	5	1:10	27.2	42.7
2	50	48	5	1:15	26.2	41.7
3	40	48	5	1:10	24.2	35.1
4	40	72	10	1:10	23.6	84.6
5	50	48	5	1:5	25.9	10.8
6	50	48	10	1:10	22.9	73.4
7	50	48	10	1:10	23.3	61.5
8	60	48	15	1:10	22.9	50.2
9	50	24	10	1:5	26.0	39.5
10	60	24	10	1:10	25.2	42.9
11	40	48	15	1:10	19.9	50.4
12	60	72	10	1:10	23.9	50.6
13	50	24	10	1:15	25.2	92.0
14	50	48	15	1:15	20.8	79.7
15	60	48	5	1:10	25.2	26.0
16	40	48	10	1:15	21.1	75.7
17	50	48	10	1:10	22.6	74.1
18	50	72	15	1:10	23.2	66.8
19	50	72	10	1:15	28.9	62.5
20	50	48	15	1:5	22.3	51.0
21	60	48	10	1:15	23.7	52.0
22	50	72	10	1:5	24.3	48.2
23	50	24	15	1:10	23.9	82.1
24	50	24	5	1:10	29.2	17.4
25	40	48	10	1:5	22.6	37.6
26	40	24	10	1:10	23.7	48.5
27	60	48	10	1:5	24.2	28.8

**Table 3:** Analysis of Variance (ANOVA) of the quadratic Box-Behnken model for monitoring the GLA + SDA proportion in the resulting LPC. Df: degrees of freedom.

Source	Sum of Squares	Df	Mean Square	F-Value	*p*-Value
Model	111.75	14	7.98	7.85	0.0005
A—Temperature	8.40	1	8.40	8.26	0.0140
B—Time	0.3605	1	0.3605	0.3547	0.5625
C—Lipase load	51.38	1	51.38	50.55	<0.0001
D—Substrate molar ratio	0.0420	1	0.0420	0.0413	0.8423
AB	0.4096	1	0.4096	0.4030	0.5375
AC	0.9801	1	0.9801	0.9643	0.3455
AD	0.2704	1	0.2704	0.2660	0.6154
BC	0.3906	1	0.3906	0.3843	0.5469
BD	7.16	1	7.16	7.04	0.0211
CD	0.8649	1	0.8649	0.8509	0.3745
A^2^	2.94	1	2.94	2.89	0.1147
B^2^	27.42	1	27.42	26.98	0.0002
C^2^	1.85	1	1.85	1.82	0.2024
D^2^	2.23	1	2.23	2.19	0.1646
Residual	12.20	12	1.02		
Lack of fit	11.92	10	1.19	8.46	0.1103
Pure error	0.2817	2	0.1408		

**Table 4:** Analysis of Variance (ANOVA) of the quadratic Box-Behnken model for monitoring LPC synthesis yield. Df: degrees of freedom.

Source	Sum of Squares	Df	Mean Square	F-Value	*p*-Value
Model	10,219.95	14	730.00	8.00	0.0005
A—Temperature	552.16	1	552.16	6.05	0.0300
B—Time	90.75	1	90.75	0.9945	0.3383
C—Lipase load	3553.52	1	3553.52	38.94	<0.0001
D—Substrate molar ratio	2935.94	1	2935.94	32.17	0.0001
AB	201.64	1	201.64	2.21	0.1629
AC	19.80	1	19.80	0.2170	0.6497
AD	55.50	1	55.50	0.6082	0.4506
BC	412.09	1	412.09	4.52	0.0550
BD	364.81	1	364.81	4.00	0.0687
CD	1.21	1	1.21	0.0133	0.9102
A^2^	864.73	1	864.73	9.48	0.0096
B^2^	3.48	1	3.48	0.0382	0.8483
C^2^	1416.29	1	1416.29	15.52	0.0020
D^2^	349.56	1	349.56	3.83	0.0740
Residual	1095.01	12	91.25		
Lack of fit	994.72	10	99.47	1.98	0.3814
Pure error	100.29	2	50.14		

**Table 5:** FA profile (expressed as percentage of total FA) and synthesis yield of LPC (mol%) obtained using either the *E. plantagineum* seed oil hydrolysate or the GLA + SDA concentrate as substrates. In both cases, reactions were carried out at 50 °C for 24 h, with a GPC:FFA molar ratio of 1:15, and a lipase load of 15 wt%, using Lipozyme^®^ 435 as the biocatalyst.

Fatty Acids	*E. plantagineum* Oil Hydrolysate	LPC	GLA + SDA Concentrate	LPC
C16:0	6.4	9.0 ± 0.1	n.d.	n.d.
C18:0	3.1	5.0 ± 0.3	n.d.	n.d.
C18:1 n-9	16.2	18.6 ± 0.5	0.7	0.6 ± 0.1
C18:2 n-6	16.7	15.0 ± 0.1	9.9	9.6 ± 0.1
C18:3 n-6 (GLA)	11.5	11.8 ± 0.4	33.3	32.8 ± 1.2
C18:3 n-3	33.9	30.0 ± 0.5	19.9	20.2 ± 0.5
C18:4 n-3 (SDA)	12.1	10.1 ± 0.2	36.2	36.7 ± 1.2
LPC yield (mol%)		89.8 ± 2.3		92.0 ± 4.1

## Data Availability

The original contributions presented in this study are included in the article/Supplementary Materials. Further inquiries can be directed to the corresponding author.

## Supplementary Materials

The following supporting information can be downloaded at: https://www.mdpi.com/article/10.3390/foods15162914/s1. Figure S1: Thin layer chromatography of crude reaction products prior to solid-phase extraction (SPE) fractionation (A); FFA recovered from the SPE cartridge and eluted with acetone:methanol 9:1 (*v*/*v*) (B); LPC-enriched fraction recovered from the SPE cartridge and eluted with methanol (C). Figure S2: Reaction scheme for LPC synthesis via esterification of glycerophosphocholine (GPC) with free fatty acids (FFA), showing the structure of the target product (*sn*-1-acyl-2-lyso-PC) together with side-products (*sn*-1-lyso-2-acyl-PC and diacylated PC) identified in the ^31^P-NMR spectrum of the LPC-enriched fraction recovered from the SPE cartridge and eluted with methanol.
