# Supplementary material for: Enzymatic Synthesis of Lysophosphatidylcholine Containing γ-Linolenic and Stearidonic Acids in a Solvent-Free System
Source: Foods. 2026 Aug 20;15(16):2914. doi: 10.3390/foods15162914 (PMC13512510; doi:10.3390/foods15162914)
Supplement: Supplementary file 1 [file foods-15-02914-s001.zip › foods-4452038-supplementary.pdf]

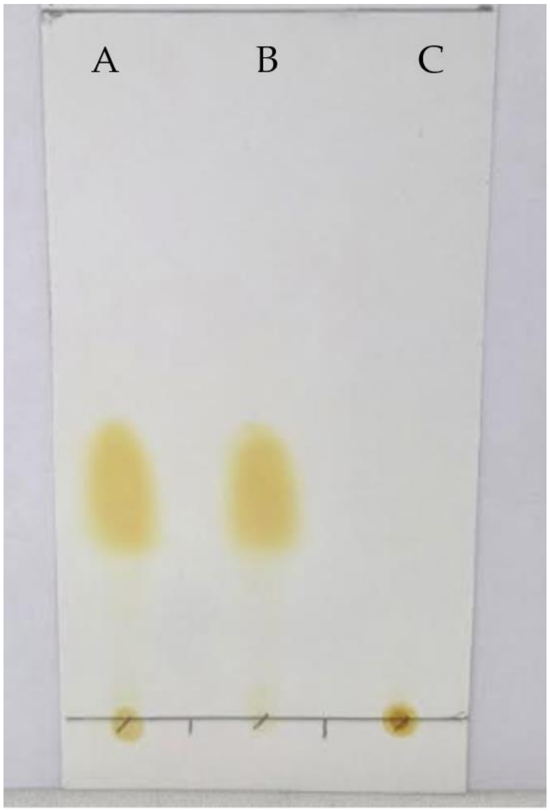

Figure S1. Thin layer chromatography of crude reaction products prior to solid-phase extraction (SPE) fractionation (A); FFA recovered from the SPE cartridge and eluted with acetone:methanol 9:1 (v/v) (B); LPC-enriched fraction recovered from the SPE cartridge and eluted with methanol (C).

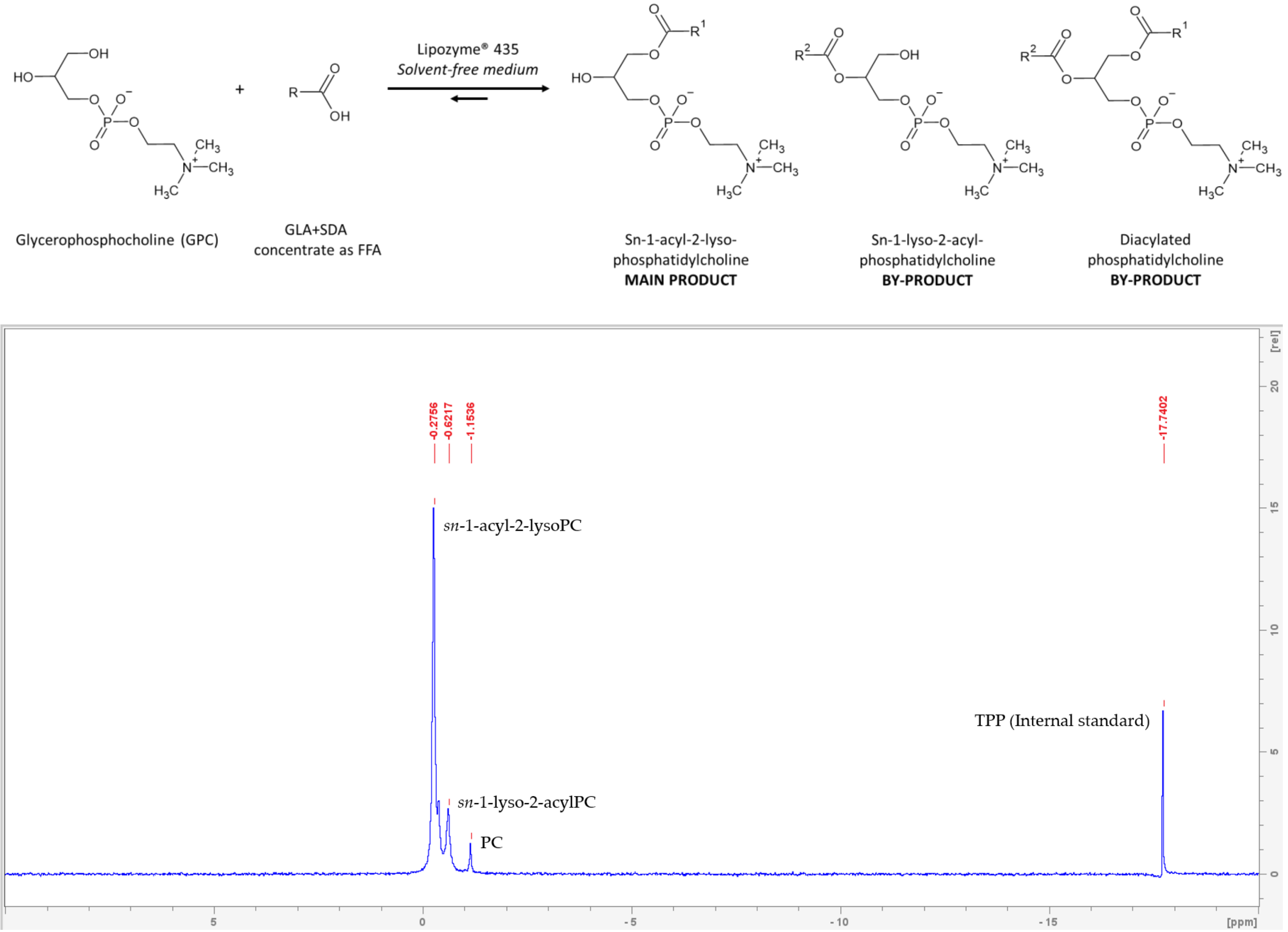

Figure S2. Reaction scheme for LPC synthesis via esterification of glycerophosphocholine (GPC) with free fatty acids (FFA), showing the structure of the target product (sn-1-acyl-2-lyso-PC) together with side-products (sn-1-lyso-2-acyl-PC and diacylated PC) identified in the ^{31}P-NMR spectrum of the LPC-enriched fraction recovered from the SPE cartridge and eluted with methanol.
